# Physical Exercise, Fitness, Cognitive Functioning, and Psychosocial Variables in an Adolescent Sample

**DOI:** 10.3390/ijerph17031100

**Published:** 2020-02-09

**Authors:** Rafael E. Reigal, Luna Moral-Campillo, Juan P. Morillo-Baro, Rocío Juárez-Ruiz de Mier, Antonio Hernández-Mendo, Verónica Morales-Sánchez

**Affiliations:** Faculty of Psychology, University of Malaga, Teatinos Campus, 29071 Malaga, Spain; rafareigal@uma.es (R.E.R.); lunamoral@uma.es (L.M.-C.); juanpablo.morillo@gmail.com (J.P.M.-B.); rjrm@uma.es (R.J.-R.d.M.); vomorales@uma.es (V.M.-S.)

**Keywords:** physical exercise, physical fitness, cognitive functioning, psychosocial well-being

## Abstract

The objective of this paper was to evaluate the relationship between physical exercise and physical fitness with cognitive and psychosocial functioning in a group of adolescents. 167 teenagers between 14 and 15 years old (*M* = 14.53; *SD* = 0.50) from the city of Malaga (Spain) participated in the study. This research used a comparative and predictive type of design. The Tanita^®^ Body Composition Monitor BC-601, some Eurofit battery tests, the D2 Attention Test, the WISC-IV Scale Symbols and Keys tests, the Form 5 Self-Concept Questionnaire (AF5), the General Self-Efficacy Scale (GSE) and the General Health Questionnaire (GHQ-28) were used to evaluate the study variables. The results found in this research pointed to a positive relationship between physical exercise and physical fitness with cognitive and psychosocial functioning in the adolescents analyzed. For example, adolescents who practiced more physical exercise had better scores on variables such as selective attention (*p* < 0.001; *η*^2^ = 0.10), processing speed (*p* < 0.001; *η*^2^ = 0.09) or general self-efficacy (*p* < 0.001; *η*^2^ = 0.15). In addition, cardiorespiratory fitness was the best predictor of test scores to assess cognitive ability and psychosocial variables. These findings suggest the need to promote physical exercise among young people because of its implications for various facets of their health and development

## 1. Introduction

Numerous studies have supported the health benefits of regular physical activity (any movement that requires energy expenditure) and exercise (physical activity that is planned, structured and repetitive) [1,2]. However, recent years have seen an increase in sedentary lifestyles among younger people, limiting the potential effects of active lifestyles [3,4]. Moreover, recent research has indicated that children and adolescents should perform physical exercise of intensity and volume from moderate to high, which only occurs in a low percentage of the population [5].

Specifically, there has been a notable increase in studies that have analyzed the link between physical exercise and cognitive functioning for this age group [6,7]. In the set of studies carried out, those that have highlighted positive relationships of physical exercise with cognitive abilities, such as attention and concentration, processing speed, cognitive functioning or language, stand out [7,8,9,10,11]. Different advances in neuroscientific knowledge help to explain why this happens, thus endorsing this phenomenon and considering it as an important line of research in the field of health [12,13].

Specifically, selective attention and concentration, as well as the speed of cognitive processing, are some of the capacities that have been studied in this age group, observing relations between these abilities and the practice of physical exercise, as well as the level of physical fitness [14,15,16,17,18,19,20,21,22]. In these and other studies that have analyzed this phenomenon, it has been emphasized that it is necessary to carry out exercise of intensity and frequency from moderate to high to increase the degree of physical fitness [23,24]. In fact, one of the best indicators to assess the relationships between these variables is the level of cardiorespiratory fitness, which has been presented as a good predictor of cognitive functioning at these ages [20,21,25].

These findings have a great impact on adolescence because of their consequences on the development of children and adolescents. In fact, adequate cognitive functioning is considered to allow for better psychosocial adjustment at these ages, facilitating adaptation to the environment and contributing to greater success in multiple academic or social tasks [26,27,28,29].

On the other hand, the regular practice of physical and sporting activity, carried out in appropriate social contexts, has been positively associated with other psychological parameters that could also have a favorable impact on child and adolescent development [26,27,28,29,30]. Thus, physical exercise usually occurs in environments that require the development of psychosocial skills necessary to function properly in them, which facilitates their training and learning, while contributing to interpersonal bonds or to the development of personal and social identity [2,26,31,32]. For this reason, the regular practice of exercise could contribute to improving the psychosocial functioning of children and adolescents, influencing aspects such as perceptions of self-concept, personal effectiveness or the assessment of one’s own health [33,34].

Self-concept is considered the perception that people have of themselves, and nowadays it is usually analyzed as a multidimensional construct constituted by perceptions of academic or labor type, familiar, social, emotional or physical self-concept, among others [35,36]. The perception of self-efficacy refers to the assessment of the capacity to successfully carry out a task [37,38]. Although it is considered that self-efficacy can be studied specifically in different contexts, some authors have developed positions that point to the possibility of assessing general self-efficacy, which would allude to a broad perception of effectiveness over a wide set of situations [39,40,41]. Finally, health perception includes judgments made about one’s physical or mental health, which is considered a strong indicator of psychosocial well-being and a predictor of future health levels [42,43]. Previous studies have associated the practice of physical exercise and a better level of physical fitness with better perceptions of self-concept, general self-efficacy and health perception in young populations [30,42,44,45,46,47,48,49].

With the purpose of analyzing the relationship between these variables, and given that few studies have addressed all of them together, the objective of this research was to analyze the relationship between physical exercise and physical fitness with cognitive and psychosocial functioning in an adolescent sample.

## 2. Materials and Methods

### 2.1. Design

A comparative and predictive design was used to carry out this research [47].

### 2.2. Participants

In total, 167 adolescents were included from the city of Malaga (Spain) (boys = 48.50%, girls = 51.50%), with ages between 14 and 15 years (*M* ± *SD*: age = 14.53 ± 0.50 years; height = 167.02 ± 10.14 cm; weight = 64.27 ± 14.75 kg; BMI = 22.95 ± 4.32 kg·m^2^). Age was used as the inclusion criterion, and those adolescents who were 14 or 15 years old during the research period were accepted for the study. Those who had a health problem that could affect the results of the study, did not perform the tests properly, or did not provide informed consent signed by their parents or legal guardians were excluded from the study.

### 2.3. Materials and Measures

#### 2.3.1. Attention Test d2 

This instrument was used to assess selective attention and concentration [50]. It is made up of a total of 658 items distributed in 14 rows. Each contains the letter “d” or “p”, which may be accompanied by one or two stripes at the top, bottom, or both. The “d” must be marked when they have two stripes (regardless of their position), considered objective stimuli. A maximum of 20 s can be spent on each row, and the order should be left to right and top to bottom. The following scores can be obtained: TA (total number of attempts), TH (total number of hits), O (omissions or number of relevant stimuli not crossed out), C (omissions or errors), TOT [total effectiveness in the test = TA − (O + C)], CON (concentration = TH − C), and VAR [index of variation between the last stimulus analyzed between different rows = (TA+) – (TA−)]. TA+ is the last stimulus analyzed in the row with the most attempted elements, and TA− is the last stimulus analyzed in the row with the fewest attempted elements.

#### 2.3.2. Wechsler Intelligence Scale Key Test and Symbol Search for Children (WISC-IV) 

These tests mainly analyze processing speed, but also attention or cognitive flexibility [51]. In the Keys Test, you must copy a series of symbols associated with a specific number. In the Symbols Test, it is necessary to decide whether there is a matching group of symbols between two groups of symbols. 120 s are available for each test. In addition, a Processing Speed Index can be extracted from the scores obtained.

#### 2.3.3. Self-Concept Form-5 Questionnaire (AF5) 

This instrument is made up of 30 items, and five dimensions of self-concept can be differentiated: academic, social, emotional, family and physical [52]. Each item is scored from 1 (total disagreement) to 99 (total agreement), and then transformed into a score between 1 and 10. The internal consistency values (Cronbach’s Alpha) in this research have been: academic = 0.80, social = 0.75, emotional = 0.72, family = 0.81, and physical = 0.83.

#### 2.3.4. General Self-Efficacy Scale (GSE) 

This questionnaire is composed of 10 items and evaluates a single dimension, the perception of competence to tackle a broad set of tasks [40,41,53,54]. Scores are from 1 (strongly disagree) to 10 (strongly disagree). The internal consistency value (Cronbach’s Alpha) in this research has been 0.82.

#### 2.3.5. General Health Questionnaire in Its 28-Item Version (GHQ-28) 

This instrument consists of 28 items and 4 dimensions: somatic symptoms, anxiety and insomnia, social dysfunction and severe depression [55,56]. We respond with scores from 0 (absence of health problems) to 3 (presence of health problems). The values of internal consistency (Cronbach’s Alpha) in this research have been: somatic symptoms = 0.79, anxiety and insomnia = 0.75, social dysfunction = 0.77 and severe depression = 0.82.

#### 2.3.6. Anthropometric and Physical Fitness Measurements

The participant’s height was evaluated using a conventional measuring rod. The weight and percentage of fat mass was analyzed with a bioimpedanciometer (Tanita^®^ Body Composition Monitor BC-601, Corp., Tokyo, Japan). The explosive force in the undercarriage was measured by means of the horizontal jump test [57]. The speed was analyzed with the test 5 × 10 m [57]. The Course Navette test [57,58] was used to evaluate maximum oxygen consumption indirectly, using the following formula: VO2max = 31.025 + 3.238S − 3.248E + 0.1536SA (where S is the maximum speed reached and A is the age of the participant).

#### 2.3.7. “Ad hoc” Questionnaire on Weekly Physical Exercise Volume

A simple questionnaire was used to collect information on how many days a week and for how long participants engaged in regular physical exercise. This questionnaire raised three aspects: (a) Do you exercise regularly? (b) How many days a week do you exercise regularly? (c) How much time do you usually spend in your physical exercise sessions?

### 2.4. Procedure

The sample was selected in different schools in the city of Malaga (Spain). Permission was first sought from the management of the center, and subsequently informed consent was obtained from parents or legal guardians. In addition, the ethical principles of the Declaration of Helsinki [59] were respected and it was approved by the Ethics Committee of the University of Malaga (No. 243, CEUMA Registry No. 18-2015-H). 

Evaluations were conducted on three different days. First, anthropometric and body composition measurements were evaluated. Second, cognitive functioning tests were performed. Finally, the psychosocial functioning questionnaires were completed and information was collected on the number of weekly hours dedicated to physical exercise, using a simple questionnaire. Based on this information, the sample was divided into three groups. Group 1 (*n* = 60; 26 boys, 34 girls) did not engage in weekly physical exercise or it was occasional (<120 min per week); group 2 (*n* = 56; 21 boys, 35 girls) engaged in regular physical exercise between 120 and 240 min per week; group 3 (*n* = 51; 34 boys, 17 girls) engaged in physical exercise more than 240 min per week.

### 2.5. Data Analysis

Descriptive and inferential analyses were performed. The Kolmogorov–Smirnov test was applied to analyze normality. In order to make comparisons between the groups, variance analysis (ANOVA), Bonferroni’s statistic and Cohen’s *d* were used to estimate the size of the effect between groups (≈0.20: small, ≈0.50: medium, and ≈0.80: large [60]). Correlations were assessed with the Pearson coefficient (±0.01 to ±0.19 = very weak correlation; ±0.20 to ±0.39 = weak correlation; ±0.40 to ±0.59 = moderate correlation; ±0.60 to ±0.79 = high correlation [61]). A multiple comparison adjustment (Bonferroni) was performed in the correlation analysis. The predictive capacity of the physical fitness over the other variables was verified by means of linear regression analysis (successive steps) [62]. The level of significance was set to α = 0.05. Analysis of variance components and generalizability analysis have also been performed to show that the sample is reliable and the results are generalizable. The software SPSS v.20.0 (IBM Corp., Armonk, NY, USA), SAS v.9.1 (SAS Institute Inc., Cary, NC, USA) [63,64] and SAGT v.1.0 (University of Malaga, Malaga, Spain) [65] were used for statistical processing.

## 3. Results

### 3.1. Descriptive Analysis and Data Normality

Table 1 and Table 2 show the descriptive and normal statistics for the total sample and for the groups constituted according to the volume of weekly physical exercise. 

### 3.2. Differences among Groups

The ANOVAs performed showed differences between the three groups in percentage of fat mass (*F*_[2,164]_ = 37.70; *p* < 0.001; *η*^2^ = 0.32; *1*−*β* = 0.99), horizontal jump (*F*_[2,164]_ = 22.87; *p* < 0.001; *η*^2^ = 0.22; *1*−*β* = 0.99), VO2max (*F*_[2,164]_ = 30.70; *p* < 0.001; *η*^2^ = 0.27; *1*−*β* = 0.99), 5x10 speed test (*F*_[2,164]_ = 14.47; *p* < 0.001; *η*^2^ = 0.15; *1*−*β* = 0.99), D2-TA (*F*_[2,164]_ = 6.63; *p* < 0.01; *η*^2^ = 0.08; *1*−*β* = 0.91), D2-TH (*F*_[2,164]_ = 7.39; *p* < 0.001; *η*^2^ = 0.08; *1*−*β* = 0.94), D2-C (*F*_[2,164]_ = 5.64; *p* < 0.01; *η*^2^ = 0.06; *1*−*β* = 0.86), D2-TOT (*F*_[2,164]_ = 8.67; *p* < 0.001; *η*^2^ = 0.10; *1*−*β* = 0.97), D2-CON (*F*_[2,164]_ = 7.70; *p* < 0.001; *η*^2^ = 0.09; *1*−*β* = 0.95), D2-TA− (*F*_[2,164]_ = 8.59; *p* < 0.001; *η*^2^ = 0.10; *1*−*β* = 0.97), Symbols (*F*_[2,164]_ = 6.09; *p* < 0.01; *η*^2^ = 0.07; *1*−*β* = 0.88), Keys (*F*_[2,164]_ = 5.37; *p* < 0.01; *η*^2^ = 0.06; *1*−*β* = 0.84), Processing Speed (*F*_[2,164]_ = 8.03; *p* < 0.001; *η*^2^ = 0.09; *1*−*β* = 0.95), academic self-concept (*F*_[2,164]_ = 3.18; *p* < 0.05; *η*^2^ = 0.04; *1*−*β* = 0.60), emotional self-concept (*F*_[2,164]_ = 8.66; *p* < 0.001; *η*^2^ = 0.10; *1*−*β* = 0.97), physical self-concept (*F*_[2,164]_ = 9.98; *p* < 0.001; *η*^2^ = 0.11; *1*−*β* = 0.98), general self-efficacy (*F*_[2,164]_ = 14.37; *p* < 0.001; *η*^2^ = 0.15; *1*−*β* = 0.99), somatic symptoms (*F*_[2,164]_ = 9.29; *p* < 0.001; *η*^2^ = 0.10; *1*−*β* = 0.99) and anxiety and insomnia (*F*_[2,164]_ = 11.24; *p* < 0.001; *η*^2^ = 0.12; *1*−*β* = 0.99). Levene’s test indicated that there was homogeneity between group variances in each case (*p < 0.*05). Table 3 shows the differences by pairs (Bonferroni) and the size of the effect (Cohen’s *d*).

### 3.3. Correlations and Linear Regression

Table 4 and Table 5 show the correlations between measures of physical fitness and cognitive and psychosocial functioning. As can be observed, there were multiple significant relationships between the variables, highlighting those established with the general indices of test d2 (TOT and CON), as well as with the measures of processing speed of the WISC-IV; also noteworthy are those established with emotional and physical self-concept, general self-efficacy, and the somatic symptom factors, anxiety, and insomnia of GHQ-28. Overall, maximum oxygen consumption should be indicated as the physical fitness measure that best correlated with the rest of the variables.

Table 6 shows the linear regression analyses (successive steps). The physical fitness measurements have been predictive and the others have been established as criteria. All models meet the assumptions of linearity in the relationship between predictor variables and criteria, homoscedasticity and normal waste distribution (mean value = 0; standard deviation = 0.99). In addition, the Durbin-Watson was between 1.56 and 1.88, being suitable according to Pardo and Ruiz [66]. As can be seen, maximum oxygen consumption was shown to be the best predictor of the cognitive and psychosocial functioning variables analyzed.

### 3.4. Generalizability Analysis

In order to verify the reliability and generalizability of the numerical structure of the sample, analysis of variance components and generalizability analysis were performed.

An analysis of variance components was performed using a least-squares procedure (VARCOM Type I) and a maximum likelihood procedure (GLM). This analysis used a 6-faceted model [y = p s e r c f], where: Participant (p) × Gender (s) × Age (e) × Exercise (r) × Instruments (c) × Factor (f). Due to the saturation produced by working with such a high number of facets, initially the model [y = p s e r c f] was used without interactions. It was obtained that the error variance with both procedures is equal (GLM = 323,117.4491/VARCOMP = 323,117), the model and the facets [p] and [f] are significant (<0.0001) and explains 48% of the variance. The rest of the facets collapsed because of the contribution to the model of the facet [p]. Next, another analysis without interactions with the model [y = s e r c f] is made to know the contribution of each facet, dispensing with the facet [p]. The model and all of the facets are significant (with the exception of the facet (c)). The model explains 16.32% of the variance.

The error variance with both procedures is equal (GLM = 520,664/VARCOMP = 520,663.5582). From this analysis, the 4 facets that contribute most variance to the model are considered, and a new analysis is performed, with all interactions, with the model [y = s|e|r|f]. The model and all the facets with their interactions are significant (with the exception of the interactions s × e × r, s × f, e × f, s × e × f, s × r × f, and, e × r × f). The model explains 22.37% of the variance. The error variance with both procedures is equal (GLM and VARCOMP). With these estimated results on the equality in the error variance, both a least-squares and a maximum likelihood procedure can be assumed that the sample is linear, normal, and homocedastic [67,68].

Using the sum of squares of these results, two generalizability analyses are performed. One with a cross-faceted design on the model [f]/[p] obtains generalizability coefficients higher than 0.95 (relative G = 0.973 and absolute G = 0.959). This data confirms the reliability and capacity of generalization of the numerical structure of the sample studied. In addition, another analysis is carried out using the [e] [r] [f]/[s] model and its different variants (Table 7). Once again, the reliability and capacity of generalization of the sample studied is confirmed.

## 4. Discussion

The aim of this research was to explore the relationships between physical exercise and physical fitness with cognitive and psychosocial functioning in an adolescent sample. To this end, groups of adolescents have been compared according to the amount of weekly exercise carried out and measures of physical fitness have been related to the cognitive and psychosocial variables under study. The results found show relations between these variables, being more favorable for those participants who performed more physical exercise and that had better levels of physical fitness, which satisfies the research objective and is congruent with previous studies [6,7,26,30].

On the one hand, teenagers who engaged in more physical exercise per week had better scores on selective attention, concentration and processing speed. These data are in line with those obtained in other studies, and also suggest that it is necessary to significantly increase physical exercise to improve results [14,15,16,18,19,22]. In fact, a greater difference has been observed between the group with the greatest and least physical exercise, than with the group with the moderate practice. Likewise, it can be observed that the group that practiced more hours of physical exercise per week had greater physical fitness. This is consistent with the postulates of several studies that indicated that increased physical fitness was an indicator of the impact that exercise has on the brain development of children and adolescents [20,21,23,24].

In recent years there have been great advances in neuroscientific knowledge applied to sports sciences, obtaining data that support how organic changes produced by physical exercise of intensity and frequency from moderate to high, would also explain the changes produced in brain functioning [12,13,24]. Therefore, it is consistent to consider that adolescents who regularly practiced more hours a week will have generated previous changes in their brain that will have helped them develop greater cognitive capacity, which has been reflected in the data obtained. Thus, as previous studies support, also reflected in this research, there has been a positive relationship in physical fitness and tests of attention, concentration and processing speed. Specifically, cardiorespiratory fitness has been the best predictor of these variables, which is consistent with previous findings [20,21,25]. Some studies have revealed that those exercises that allow to increase cardiorespiratory fitness, also generate increases in the synthesis of biomolecules such as the brain-derived neurotrophic factor (BDNF) or the insulin-like growth factor-1 (IGF-1), which facilitate the brain plasticity processes [13]. In turn, it has been observed that cardiovascular fitness is positively associated with the cortical and subcortical gray matter volume in various brain regions [24].

On the other hand, it has also been observed in this study that adolescents who performed more hours of physical exercise per week and had better physical fitness scored better in some dimensions of self-concept, had better perception of general self-efficacy and health. In addition, cardiorespiratory fitness would again stand out as the best predictor of these variables. These data are congruent with other research that had highlighted these partnerships [42,44,45,46,47,48,49,69], and contributes to consolidating the positive relationship between physical exercise and various psychological variables that may contribute to a better psychosocial development of people at these ages [26,30,33,34].

There are many factors that can modulate the relationships between exercise and psychosocial development. Although the type of design used in this research cannot explain causal relationships, it is considered that adequate contexts of physical exercise would generate environments in children and adolescents in which they have to overcome challenges and adapt to different situations, they could acquire greater sensations of competition, a better perception of body image or of their state of health [2,28,31,32]. In other words, exercise would contribute to acquiring a series of personal and social skills that would constitute an extrapolable advantage to other contexts, improving their psychosocial aptitude and capacity to adapt to the continuous demands of their social environment [26,27].

This paper has some limitations. The type of design does not facilitate causal explanations, so longitudinal or quasi-experimental designs could be used in future work. Additionally, it would be interesting to collect information on the intensity and type of physical exercise, as well as the amount of physical activities, information about the family, financial background, parental education, etc., in order to perform analyzes based on these variables. These parameters could generate essential information to understand how physical exercise is related to the measures analyzed in the present study. Likewise, aspects such as the family environment, the time they use to study or sleep, could be analyzed due to the possible influence they might have on the results. Furthermore, it would be appropriate to analyze whether the gender variable is a factor that could condition the results found. In addition, it would be interesting to analyze the interactions between the variables of cognitive and psychosocial functioning, to observe how they modulate among themselves their relationships with the practice of exercise.

However, this work represents an interesting approximation to the joint study of these variables, which had hardly been previously addressed, and allows us to obtain a broader perspective of the relations between physical exercise and physical fitness with the rest of the measures under study. In addition, effect size calculations are included in most analyses, which is relevant for estimating the magnitude of differences between the groups analyzed. Therefore, this work provides valuable information in this field of knowledge, and its data suggest the need to widely promote physical exercise among the youngest because of its involvement in issues relating to their health and their integral development.

## 5. Conclusions

This study highlights the relationships among physical exercise, physical fitness, cognitive and psychosocial functioning in an adolescent sample. On the one hand, it shows that adolescents who practice exercise regularly have better scores on selective attention and processing speed, as well as better self-concept, self-efficacy and self-perceived health. In addition, this research points out that it is necessary to increase the levels of physical fitness so that the relationships between physical exercise and health are more evident. Therefore, the results of this research suggest the need to continue promoting physical activity and exercise at these ages, due to its impact on the health and development of adolescents.

## Figures and Tables

**Table 1:** Mean and standard deviation of study variables.

	Total (*n* = 167)		Group 1 (*n* = 60)		Group 2 (*n* = 56)		Group 3 (*n* = 51)	
	*M*	*SD*	*M*	*SD*	*M*	*SD*	*M*	*SD*
% FM	22.54	9.57	28.73	8.85	22.25	7.20	15.57	7.66
HJT	161.08	39.91	141.35	32.03	158.71	35.54	186.90	39.18
VO2max	41.46	8.79	36.21	7.12	41.61	7.54	47.46	8.02
5 × 10	19.01	2.23	19.95	2.09	19.09	2.16	17.84	1.94
D2-TA	60.55	18.98	54.52	18.40	60.93	19.01	67.24	17.59
D2-TH	60.77	20.60	53.50	19.61	62.11	19.39	67.84	20.61
D2-O	46.69	21.32	43.22	18.96	49.77	23.95	47.41	20.68
D2-C	47.24	16.74	41.63	16.96	49.73	16.97	51.10	14.63
D2-TOT	60.77	18.72	53.97	17.66	61.36	18.28	68.14	17.80
D2-CON	59.91	20.88	52.28	19.75	61.64	19.76	66.98	20.82
D2-TA+	55.49	16.26	53.10	19.15	55.45	14.61	58.35	13.96
D2-TA−	64.02	19.05	57.08	17.26	64.71	19.71	71.43	17.67
D2-VAR	44.99	19.59	47.47	21.16	44.95	18.65	42.14	18.65
SIM	11.36	2.67	10.65	3.00	11.21	2.26	12.35	2.41
CL	10.19	3.24	9.22	3.14	10.32	2.82	11.18	3.53
VP	105.35	13.23	100.82	14.57	105.50	10.28	110.51	12.76
AF5-A	5.74	1.69	5.43	1.64	5.63	1.56	6.21	1.80
AF5-S	6.03	1.04	6.15	0.89	5.90	1.02	6.05	1.22
AF5-E	6.26	1.79	5.70	1.84	6.13	1.59	7.05	1.70
AF5-F	6.36	0.91	6.32	0.81	6.41	0.92	6.36	1.01
AF5-P	5.83	1.96	5.24	2.07	5.62	1.80	6.77	1.63
GSE	6.88	2.06	5.95	2.30	6.93	1.49	7.90	1.82
GHQ-SS	0.75	0.46	0.83	0.50	0.87	0.42	0.53	0.37
GHQ-AI	0.92	0.74	1.09	0.75	1.07	0.73	0.53	0.56
GHQ-SDy	0.93	0.47	0.99	0.55	0.98	0.47	0.82	0.37
GHQ-SDe	0.45	0.55	0.48	0.60	0.54	0.53	0.31	0.46

*Note*: % FM = Percentage of body fat mass; HJT = Horizontal jump test (cm); 5 × 10 = Speed test (sec); VO2max = Maximum oxygen consumption (mL/kg/min); D2 = Test D2; TA = Total number of attempts; TH = Total hits; O = Omissions; C = Commissions; TOT = Total effectiveness in the test; CON = Concentration index; VAR = Variation index; (TA+) = Line with the greatest number of attempted elements; (TA−) = Line with fewer elements attempted; SIM = Symbols; CL = Keys; VP = Processing Speed; AF5 = Self-Concept Form-5; A = Academic; S = Social; E = Emotional; F = Family; P = Physical; GSE = General Self-efficacy; GHQ = Health perception questionnaire; SS = Somatic symptoms; AI = Anxiety and insomnia; SDy = Social dysfunction; SDe = Severe Depression.

**Table 2:** Skewness, curtosis and Kolmogorov–Smirnov (*Z*) of study variables.

Study Variables	Total (*n* = 167)			Group 1 (*n* = 60)			Group 2 (*n* = 56)			Group 3 (*n* = 51)		
	*S*	*K*	*Z*	*S*	*K*	*Z*	*S*	*K*	*Z*	*S*	*K*	*Z*
% FM	0.03	−1.18	1.23	−0.75	−0.22	1.25	−0.18	−1.01	1.09	0.86	−0.70	1.18
HJT	0.45	−0.71	1.17	1.34	2.27	1.31	0.24	−0.68	0.89	−0.25	−0.81	0.69
VO2max	0.36	−1.48	1.24	1.54	1.02	1.07	0.51	−1.35	0.95	−0.71	−0.91	1.22
5 × 10	0.32	−0.84	0.98	−0.19	−0.15	0.56	0.23	−0.95	0.88	1.35	1.31	1.17
D2-TA	0.09	−1.21	1.14	0.74	−0.74	1.27	−0.01	−1.07	0.78	−0.49	−0.58	1.15
D2-TH	−0.18	−1.14	1.02	0.41	−0.95	1.14	−0.27	−0.58	1.01	−0.86	−0.49	1.06
D2-O	0.51	−0.06	1.18	0.49	0.36	0.85	0.38	−0.54	0.89	0.53	0.19	0.65
D2-C	−0.02	−0.71	1.33	0.09	−0.91	1.12	0.04	−0.43	1.18	0.00	−1.23	1.04
D2-TOT	−0.01	−1.45	1.30	0.77	−0.92	1.19	−0.14	−1.18	1.11	−0.79	−0.62	1.26
D2-CON	−0.12	−1.24	1.21	0.48	−0.93	0.97	−0.14	−0.89	1.16	−0.83	−0.57	1.18
D2-TA+	−0.03	−0.32	1.35	0.38	−0.58	0.80	0.06	0.14	0.89	−0.87	0.48	1.22
D2-TA−	−0.16	−1.23	1.16	0.48	−0.72	1.15	−0.31	−1.01	1.23	−0.84	−0.58	1.16
D2-VAR	−0.09	−0.59	0.81	−0.04	−0.90	0.95	−0.05	−0.32	0.87	−0.40	−0.58	0.81
SYM	0.31	0.00	1.36	0.28	−0.76	0.97	0.54	−0.10	0.91	0.84	1.06	1.25
KEY	0.36	0.18	1.04	0.12	−0.12	1.04	0.35	1.05	1.20	0.51	−0.61	1.30
PS	0.15	−0.18	1.10	0.30	−0.40	0.65	0.18	0.47	0.77	0.33	−0.78	0.76
AF5-A	0.07	−0.80	0.95	−0.19	−0.72	0.76	0.33	−0.31	1.01	−0.03	−1.20	0.85
AF5-S	−1.55	1.11	1.12	−0.31	1.01	0.99	−1.93	1.52	1.18	−1.86	1.37	1.21
AF5-E	−0.23	−1.00	1.26	−0.06	−1.31	0.98	0.06	−1.19	1.12	−0.81	0.27	0.92
AF5-F	−0.51	1.46	0.96	0.02	0.76	0.95	−0.04	−0.34	0.61	−1.26	1.23	1.03
AF5-P	−0.22	−0.47	0.83	−0.30	−0.53	0.64	0.28	−0.71	0.85	−0.18	−0.98	0.70
GSE	−0.33	−0.32	1.08	−0.14	−1.03	1.16	0.10	−0.40	0.86	−0.23	−0.68	0.95
GHQ-SS	0.41	−0.17	1.31	0.38	0.14	0.94	0.10	−0.54	0.92	0.61	−0.60	1.26
GHQ-AI	0.63	−0.27	1.19	0.30	−0.48	0.71	0.54	−0.12	0.73	1.20	0.66	1.15
GHQ-SDy	0.94	1.68	1.24	1.08	0.82	1.17	0.77	1.44	0.85	0.03	−0.25	0.95
GHQ-SDe	1.50	1.65	1.18	1.46	1.76	1.16	1.46	1.37	1.19	1.64	1.75	1.22

*Note*: *S =* Skewness; *K =* Curtosis; *Z =* Kolmogorov-Smirnov (*Z*); % FM = Percentage of body fat mass; HJT = Horizontal jump test (cm); 5 × 10 = Speed test (sec); VO2max = Maximum oxygen consumption (ml/kg/min); D2 = Test D2; TA = Total number of attempts; TH = Total hits; O = Omissions; C = Commissions; TOT = Total effectiveness in the test; CON = Concentration index; (TA+) = Line with the greatest number of elements tried; (TA−) = Line with fewer elements attempted; VAR = Index of variation; SYM = Symbols; KEY = Keys; PS = Processing Speed; AF5 = Self-Concept Form-5; A = Academic; S = Social; E = Emotional; F = Family; P = Physical; GSE = General Self-efficacy; GHQ = Health perception questionnaire; SS = Somatic symptoms; AI = Anxiety and insomnia; SDy = Social dysfunction; SDe = Severe Depression.

**Table 3:** Differences among groups for each variable.

Study Variables	Group 1 vs. Group 2		Group 2 vs. Group 3		Group 1 vs. Group 3	
	*p*-Value	Cohen’s *d* [95% CI]	*p*-Value	Cohen’s *d* [95% CI]	*p*-Value	Cohen’s *d* [95% CI]
% FM	<0.001	−0.80 [−1.18, −0.42]	<0.001	−0.90 [−1.30, −0.50]	<0.001	−1.58 [−2.08, −1.15]
HJT	<0.01	0.51 [0.14, 0.88]	<0.001	0.76 [0.36, 1.15]	<0.001	1.28 [0.87, 1.69]
VO2max	<0.001	0.74 [0.36, 1.11]	<0.001	0.75 [0.35, 1.14]	<0.001	1.49 [1.07, 1.91]
5 × 10	<0.05	−0.40 [−0.77, −0.04]	<0.01	−0.60 [−0.99, −0.22]	<0.001	−1.04 [−1.44, −0.65]
D2-TA	*---*	*---*	*---*	*---*	<0.001	0.34 [−0.03, 0.73]
D2-TH	<0.05	0.44 [0.07, 0.81]	*---*	*---*	<0.001	0.71 [0.33, 1.10]
D2-C	<0.01	0.48 [0.11, 0.85]	*---*	*---*	<0.01	0.59 [0.21, 0.98]
D2-TOT	<0.05	0.41 [0.04, 0.78]	*---*	*---*	<0.001	0.80 [0.41, 1.19]
D2-CON	<0.05	0.47 [0.10, 0.84]	*---*	*---*	<0.001	0.73 [0.34, 1.11]
D2-TA−	<0.05	0.42 [0.06, 0.78]	*---*	*---*	<0.001	0.82 [0.43, 1.21]
SYM	*---*	*---*	<0.05	0.49 [0.10, 0.87]	<0.001	0.62 [0.24, 1.01]
KEY	*---*	*---*	*---*	*---*	<0.01	0.59 [0.21, 0.97]
PS	<0.05	0.37 [0.01, 0.74]	<0.05	0.43 [0.05, 0.82]	<0.001	0.70 [0.32, 1.09]
AF5-A	*---*	*---*	*---*	*---*	<0.05	0.34 [−0.04, 0.73]
AF5-E	*---*	*---*	<0.01	0.56 [0.17, 0.95]	<0.001	0.76 [0.37, 1.15]
AF5-P	*---*	*---*	<0.01	0.67 [0.28, 1.06]	<0.001	0.81 [0.43, 1.20]
GSE	<0.01	0.50 [0.13, 0.87]	<0.01	0.59 [0.20, 0.97]	<0.001	0.93 [0.54, 1.32]
GHQ-SS	*---*	*---*	<0.001	−0.86 [−1.25, −0.46]	<0.001	−0.67 [−1.06, −0.29]
GHQ-AI	*---*	*---*	<0.001	−0.83 [−1.22, −0.43]	<0.001	−0.84 [−1.23, −0.45]

*Note*: % FM = Percentage of body fat mass; HJT = Horizontal jump test (cm); 5 × 10 = Speed test (sec); VO2max = Maximum oxygen consumption (mL/kg/min); D2 = Test D2; TA = Total number of attempts; TH = Total hits; TOT = Total effectiveness in the test; CON = Concentration index; (TA−) = Line with fewer elements attempted; SYM = Symbols; KEY = Keys; PS = Processing Speed; AF5 = Self-Concept Form-5; A = Academic; E = Emotional; P = Physical; GSE = General Self-efficacy; GHQ = Health perception questionnaire; SS = Somatic symptoms; AI = Anxiety and insomnia.

**Table 4:** Correlation analysis among physical fitness and cognitive functioning measures.

Physical Variables	D2									WISC-IV		
	TA	TH	O	C	TOT	CON	TA+	TA−	VAR	SYM	KEY	PS
% FM	−0.24	−0.26 *	−0.10	−0.09	−0.29 **	−0.28 *	−0.15	−0.20	0.07	−0.37 ***	−0.28 **	−0.38 ***
HJT	0.25 *	0.27 *	0.12	0.07	0.30 **	0.29 **	0.19	0.18	−0.03	0.36 ***	0.27 **	0.36 ***
VO2max	0.32 ***	0.31 **	0.08	0.06	0.38 ***	0.34 ***	0.27 *	0.26 *	−0.03	0.44 ***	0.30 ***	0.43 ***
5 × 10	−0.16 *	−0.19	−0.13	−0.01	−0.22	−0.21	−0.12	−0.17 *	0.04	−0.28 **	−0.22 *	−0.29 **

*Note*: % FM = Percentage of body fat mass; HJT = Horizontal jump test (cm); 5 × 10 = Speed test (sec); VO2max = Maximum oxygen consumption (mL/kg/min); D2 = Test D2; TA = Total number of attempts; TH = Total hits; O = Omissions; C = Commissions; TOT = Total effectiveness in the test; CON = Concentration index; (TA+) = Line with the highest number of elements tried; TA− = Line with the lowest number of elements tried; VAR = Variation index; SYM = Symbols; KEY = Keys; PS = Processing speed. * *p* < 0.05; ** *p* < 0.01; *** *p* < 0.001.

**Table 5:** Correlation analysis among physical fitness and psychosocial measures.

Physical Variables	AF5					GSE	GHQ			
	A	S	E	F	P		SS	AI	SDy	SDe
% FM	−0.21	−0.05	−0.33 ***	−0.14	−0.49 ***	−0.44 ***	0.42 ***	0.34 ***	0.14	0.11
HJT	0.23	0.04	0.36 ***	0.13	0.48 ***	0.41 ***	−0.51 ***	−0.41 ***	−0.11	−0.12
VO2max	0.22	0.09	0.39 ***	0.12	0.56 ***	0.46 ***	−0.55 ***	−0.40 ***	−0.16	−0.18
5 × 10	−0.19	−0.03	−0.31 ***	−0.14	−0.38 ***	−0.29 ***	0.42 ***	0.36 ***	0.09	0.06

*Note*: % FM = Percentage of body fat mass; HJT = Horizontal jump test (cm); 5 × 10 = Speed test (sec); VO2max = Maximum oxygen consumption (mL/kg/min); AF5 = Self-Concept Form-5; A = Academic; S = Social; E = Emotional; F = Family; P = Physical; GSE = General Self-efficacy; GHQ = Health perception questionnaire; SS = Somatic symptoms; AI = Anxiety and insomnia; SDy = Social dysfunction; SDe = Severe depressions. *** *p* < 0.001.

**Table 6:** Linear regression analysis of D2 test factors, processing speed, self-concept (AF5), general self-efficacy (GSE) and self-related health (GHQ-28) regressed on physical fitness predictors (successive steps).

F	R^2^	D-W	Criterion	Predictor	B	t
19.36 ***	0.10	1.73	D2-TA	VO2max	0.32	4.40 ***
18.02 ***	0.09	1.61	D2-TH	VO2max	0.31	4.25 ***
27.49 ***	0.14	1.57	D2-TOT	VO2max	0.38	5.24 ***
21.63 ***	0.11	1.75	D2-CON	VO2max	0.34	4.65 ***
12.91 ***	0.07	1.68	D2-TA+	VO2max	0.27	3.59 ***
12.36 ***	0.06	1.66	D2-TA−	VO2max	0.26	3.52 ***
40.18 ***	0.19	1.56	SYM	VO2max	0.44	6.34 ***
16.76 ***	0.09	1.60	KEY	VO2max	0.30	4.09 ***
36.93 ***	0.18	1.75	PS	VO2max	0.43	6.08 ***
15.14 ***	0.08	1.68	AF5-C	HJT	0.30	3.89 ***
29.13 ***	0.15	1.82	AF5-E	VO2max	0.39	5.40 ***
73.48 ***	0.30	1.62	AF5-P	VO2max	0.56	8.57 ***
24.62 ***	0.22	1.63	GSE	VO2max	0.30	2.86 **
				% FM	−0.21	−2.03 *
70.84 ***	0.30	1.80	GHQ-SS	VO2max	−0.55	−8.42 ***
33.88 ***	0.17	1.84	GHQ-AI	HJT	−0.41	−5.82 ***
4.31 *	0.02	1.77	GHQ-SDy	VO2max	−0.16	−2.08 *
5.29 *	0.03	1.88	GHQ-SDe	VO2max	−0.18	−2.30 *

*Note*: % FM = Percentage of body fat mass; HJT = Horizontal jump test (cm); VO2max = Maximum oxygen consumption (mL/kg/min); D2 = Test D2; TA = Total number of attempts; TH = Total number of successes; TOT = Total effectiveness in the test; CON = Concentration index; TA+ = Line with the greatest number of elements tried; TA− = Line with fewer elements attempted; SYM = Symbols; KEY = Keys; PS = Processing Speed; AF5 = Self-Concept Form-5; A = Academic; E = Emotional; P = Physical; GSE = General Self-efficacy; GHQ = Health perception questionnaire; SS = Somatic symptoms; AI = Anxiety and insomnia; SDy = Social dysfunction; SDe = Severe depression. * *p* < 0.05; ** *p* < 0.01; *** *p* < 0.001.

**Table 7:** Absolute generalizability coefficient and relative generalizability coefficient for [e] [r] [f] / [s] model and its different variants.

Model	Generalizability Coefficients
[e] [r] [f]/[s]	G relative = 1
	G absolute = 1
[e] [r] [f]/[s]	G relative = 0.783
	G absolute = 0.745
[s] [e] [r]/[f]	G relative = 0.924
	G absolute = 0.819
[s] [r] [f]/[e]	G relative = 0.848
	G absolute = 0.848

s = Gender; e = Age; r = Exercise; f = Factor.

## References

[1] Biddle S., Ciaccioni S., Thomas G., Vergeer I. (2019). Physical activity and mental health in children and adolescents: An updated review of reviews and an analysis of causality. Psychol. Sport Exerc..

[2] Swann C., Telenta J., Draper G., Liddle S., Fogarty A., Hurley D., Vella S. (2018). Youth sport as a context for supporting mental health: Adolescent male perspectives. Psychol. Sport Exerc..

[3] Hynynen S.T., Van Stralen M.M., Sniehotta F.F., Araújo-Soares V., Hardeman W., Chinapaw M.J.M., Vasankari T., Hankonen N. (2016). A systematic review of school-based interventions targeting physical activity and sedentary behaviour among older adolescents. Int. Rev. Sport Exer. Psychol..

[4] Mann K.D., Howe L.D., Basterfield L., Parkinson K.N., Pearce M.S., Reilly J.K., Adamson A.J., Reilly J.J., Janssen X. (2017). Longitudinal study of the associations between change in sedentary behavior and change in adiposity during childhood and adolescence: Gateshead Millennium Study. Int. J. Obes..

[5] Hayes G., Dowd K.P., MacDonncha C., Donnelly A.E. (2019). Tracking of physical activity and sedentary behavior from adolescence to young adulthood: A systematic literature review. J. Adolesc. Health.

[6] Cooper S.B., Dring K.J., Morris J.G., Sunderland C., Bandelow S., Nevill M.E. (2018). High intensity intermittent games-based activity and adolescents’ cognition: Moderating effect of physical fitness. BMC Public Health.

[7] Xue Y., Yang Y., Huang T. (2019). Effects of chronic exercise interventions on executive function among children and adolescents: A systematic review with meta-analysis. Br. J. Sport Med..

[8] Donnelly J.E., Hillman C.H., Castelli D.M., Etnier J.L., Lee S.M., Tomporowski P.D., Lambourne K., Szabo-Reed A.N. (2016). Physical activity, fitness, cognitive function, and academic achievement in children: A systematic review. Med. Sci. Sports Exerc..

[9] Li J.W., O’Connor H., O’Dwyer N., Orr R. (2017). The effect of acute and chronic exercise on cognitive function and academic performance in adolescents: A systematic review. J. Sci. Med. Sport.

[10] Liu J.H., Alderman B.L., Song T.F., Chen F.T., Hung T.M., Chang Y.K. (2018). A randomized controlled trial of coordination exercise on cognitive function in obese adolescents. Psychol. Sport Exerc..

[11] Ludyga S., Gerber M., Herrmann C., Brand S., Pühse U. (2018). Chronic effects of exercise implemented during school-break time on neurophysiological indices of inhibitory control in adolescents. Trends Neurosci. Educ..

[12] Chaddock L., Erickson K.I., Holtrop J.L., Voss M.W., Pontifex M.B., Raine L.B., Hillman C.H., Kramer A.F. (2014). Aerobic fitness is associated with greater white matter integrity in children. Front. Hum. Neurosci..

[13] Tari A.R., Norevik C.S., Scrimgeour N.R., Kobro-Flatmoen A., Storm-Mathisen J., Bergersen L.H., Wrann C.D., Selbæk G., Kivipelto M., Moreira J.B.N. (2019). Are the neuroprotective effects of exercise training systemically mediated?. Prog. Cardiovasc. Dis..

[14] Cadenas-Sanchez C., Vanhelst J., Ruiz J.R., Castillo-Gualda R., Libuda L., Labayen I., De Miguel-Etayo P., Marcos A., Molnár E., Catena A. (2017). Fitness and fatness in relation with attention capacity in European adolescents: The HELENA study. J. Sci. Med. Sport.

[15] Cserjési R., Molnár D., Luminet O., Lénárd L. (2007). Is there any relationship between obesity and mental flexibility in children?. Appetite.

[16] Guiney H., Machado L. (2013). Benefits of regular aerobic exercise for executive functioning in healthy populations. Psychon. Bull. Rev..

[17] Hillman C.H., Castelli D.M., Buck S.M. (2005). Aerobic fitness and neurocognitive function in healthy preadolescent children. Med. Sci. Sport Exer..

[18] Hillman C.H., Kamijo K., Scudder M. (2011). A review of chronic and acute physical activity participation on neuroelectric measures of brain health and cognition during childhood. Prev. Med..

[19] Pérez-Lobato R., Reigal R.E., Hernández-Mendo A. (2016). Relationships between physical practice, physical condition, and attention in a sample of adolescents. J. Sport Psychol..

[20] Pontifex M.B., Raine L.B., Johnson C.R., Chaddock L., Voss M.W., Cohen N.J., Kramer A.F., Hillman C.H. (2011). Cardiorespiratory fitness and the flexible modulation of cognitive control in preadolescent children. J. Cogn. Neurosci..

[21] Reloba-Martínez S., Reigal R.E., Hernández-Mendo A., Martínez-López E.J., Martín-Tamayo I., Chirosa-Ríos L.J. (2017). Effects of vigorous extracurricular physical exercise on the attention of schoolchildren. J. Sport Psychol..

[22] Tine M. (2014). Acute aerobic exercise: An intervention for the selective visual attention and reading comprehension of low-income adolescents. Front. Psychol..

[23] Chaddock L., Erickson K.I., Kienzler C., Drollette E., Raine L., Kao S.C., Bensken J., Weisshappel R., Castelli D.M., Hilla¡man C.H. (2018). Physical activity increases white matter microstructure in Children. Front. Neurosci..

[24] Esteban-Cornejo I., Cadenas-Sanchez C., Contreras-Rodriguez O., Verdejo-Roman J., Mora-Gonzalez J., Migueles J.H., Henriksson P., Davis C.L., Verdejo-García A., Catena A. (2017). A whole brain volumetric approach in overweight/obese children: Examining the association with different physical fitness components and academic performance. The ActiveBrains project. NeuroImage.

[25] Herting M.M., Colby J.B., Sowell E.R., Nagel B.J. (2014). White matter connectivity and aerobic fitness in male adolescents. Dev. Cogn. Neuros..

[26] Lubans D., Richards J., Hillman C., Faulkner G., Beauchamp M., Nilsson M., Kelly P., Smith J., Raine L., Biddle S. (2016). Physical activity for cognitive and mental health in youth: A systematic review of mechanisms. Pediatrics.

[27] Perlman S.B., Hein T.C., Stepp S.D. (2014). Emotional reactivity and its impact on neural circuitry for attention–emotion interaction in childhood and adolescence. Dev. Cogn. Neuros..

[28] Rabiner D.L., Godwin J., Dodge K.A. (2016). Predicting academic achievement and attainment: The contribution of early academic skills, attention difficulties, and social competence. School Psychol. Rev..

[29] Zmyj N., Witt S., Weitkämper A., Neumann H., Lücke T. (2017). Social cognition in children born preterm: A perspective on future research directions. Front. Psychol..

[30] Utesch T., Dreiskämper D., Naul R., Geukes K. (2018). Understanding physical (in-) activity, overweight, and obesity in childhood: Effects of congruence between physical self-concept and motor competence. Sci. Rep..

[31] Holt N.L. (2008). Positive Youth Development through Sport.

[32] Marker A.M., Steele R.G., Noser A.E. (2018). Physical activity and health-related quality of life in children and adolescents: A systematic review and meta-analysis. Health Psychol..

[33] Collins H., Booth J.N., Duncan A., Fawkner S., Niven A. (2019). The effect of resistance training interventions on’the self’in youth: A systematic review and meta-analysis. Sports Med. Open.

[34] Rey O., Vallier J.M., Nicol C., Mercier C.S., Maïano C. (2018). Repeated Effects of Vigorous Interval Training in Basketball, Running-Biking, and Boxing on the Physical Self-Perceptions of Obese Adolescents. J. Appl. Sport Psychol..

[35] Esnaola I., Sesé A., Antonio-Agirre I., Azpiazu L. (2018). The Development of Multiple Self-Concept Dimensions During Adolescence. J. Res. Adolesc..

[36] Shavelson R.J., Hubner J.J., Stanton J.C. (1976). Self-concept: Validation of construct interpretations. Rev. Educ. Res..

[37] Bandura A. (1986). Social Foundations of Thought and Action: A Social Cognitive Theory.

[38] Bandura A. (1997). Self-Efficacy: The Exercise of Control.

[39] Luszczynska A., Scholz U., Schwarzer R. (2005). The general self-efficacy scale: Multicultural validation studies. J. Psychol..

[40] Schwarzer R. (1992). Self-Efficacy: Thought Control of Action.

[41] Schwarzer R., Jerusalem M., Weinman J., Wright S., Johnston M. (1995). Generalized Self-Efficacy scale. Measures in Health Psychology: A User’s Portfolio. Casual and Control Beliefs.

[42] Bombak A.E. (2013). Self-rated health and public health: A critical perspective. Front. Public Health.

[43] Torsheim T., Nygren J.M., Rasmussen M., Arnarsson A.M., Bendtsen P., Schnohr C.W., Nielsen L., Nyholm M. (2018). Social inequalities in self-rated health: A comparative cross-national study among 32,560 Nordic adolescents. Scand. J. Public Health.

[44] Christiansen L.B., Lund-Cramer P., Brondeel R., Smedegaard S., Holt A.D., Skovgaard T. (2018). Improving children’s physical self-perception through a school-based physical activity intervention: The Move for Well-being in School study. Ment. Health Phys. Act..

[45] Gall K., van Zutven K., Lindstrom J., Bentley C., Gratwick-Sarll K., Harrison C., Lewis V., Mond J. (2016). Obesity and emotional well-being in adolescents: Roles of body dissatisfaction, loss of control eating, and self-rated health. Obesity.

[46] Ho F.K.W., Louie L.H.T., Chow C.B., Wong W.H.S., Ip P. (2015). Physical activity improves mental health through resilience in Hong Kong Chinese adolescents. BMC Pediatr..

[47] Kantomaa M.T., Tammelin T., Ebeling H., Stamatakis E., Taanila A. (2015). High levels of physical activity and cardiorespiratory fitness are associated with good self-rated health in adolescents. J. Phys. Act. Health.

[48] Liu M., Wu L., Ming Q. (2015). How does physical activity intervention improve self-esteem and self-concept in children and adolescents? Evidence from a meta-analysis. PLoS ONE.

[49] Ato M., López-García J.J., Benavente A. (2013). A classification system for research designs in psychology. An. Psicol..

[50] Brickenkamp R. (2002). D-2. Attention Task.

[51] Wechsler D. (2005). Wechsler Intelligence Scale for Children (WISC-IV).

[52] García J.F., Musitu G., Veiga F. (2006). Self-concept in adults from Spain and Portugal. Psicothema.

[53] Baessler J., Schwarzer R. (1996). Evaluation of Self-Efficacy: Spanish Adaptation of the General Self-Efficacy Scale. Anxiety Stress.

[54] Sanjuán P., Pérez A.M., Bermúdez J. (2000). Escala de autoeficacia general: Datos psicométricos de la adaptación para población española. Psicothema.

[55] Goldberg D.P. (1978). Manual of the General Health Questionnaire.

[56] Lobo A., Pérez-Echeverría M.J., Artal J. (1986). Validity of escaled versión of de General Health Questionnaire (GHQ-28) in a Spanish population. Psychol. Med..

[57] Eurofit (1993). Handbook for the Eurofit Test on Physical Fitness.

[58] Léger L.A., Mercier D., Gadoury C., Lambert J. (1988). The multistage 20 metre shuttle run test for aerobic fitness. J. Sport Sci..

[59] World Medical Association (2013). World Medical Association Declaration of Helsinki: Ethical principles for medical research involving human subjects. J. Am. Med. Assoc..

[60] Hojat M., Xu G. (2004). A visitor’s guide to effect sizes: Statistical significance versus practical (clinical) importance of research findings. Adv. Health Sci. Educ. Theory Pract..

[61] Evans J.D. (1996). Straightforward Statistics for the Behavioral Sciences.

[62] Ruiz-Barquín R. (2008). Contributions of the sub-dimensional analysis of the BFQ personality questionnaire for the prediction of performance in young competitive judokas. Sport Psychol. Noteb..

[63] SAS Institute (1999). User’s Guide.

[64] Schlotzhauer S.D., Littell R. (1997). SAS System for Elementary Statistical Analysis.

[65] Hernández-Mendo A., Blanco-Villaseñor A., Pastrana J.L., Morales-Sánchez V., Ramos-Pérez F.J. (2016). SAGT: New software for generalizability analysis. Rev. Iberoam. Psicol. Ejerc. Deporte.

[66] Pardo A., Ruiz M.A. (2005). Data Analysis with SPSS 13 Base.

[67] Hemmerle W.J., Hartley H.O. (1973). Computing maximum likelihood estimates for the mixed AOV model using the W transformation. Technometrics.

[68] Searle S., Casella G., McCulloch C. (1992). Variance Components.

[69] Becerra-Fernández C.A., Reigal R.E., Hernández-Mendo A., Martín-Tamayo I. (2013). Relationships of physical condition and body composition with self-perception of health. RICYDE. Rev. Int. Cienc. Deporte.

